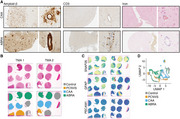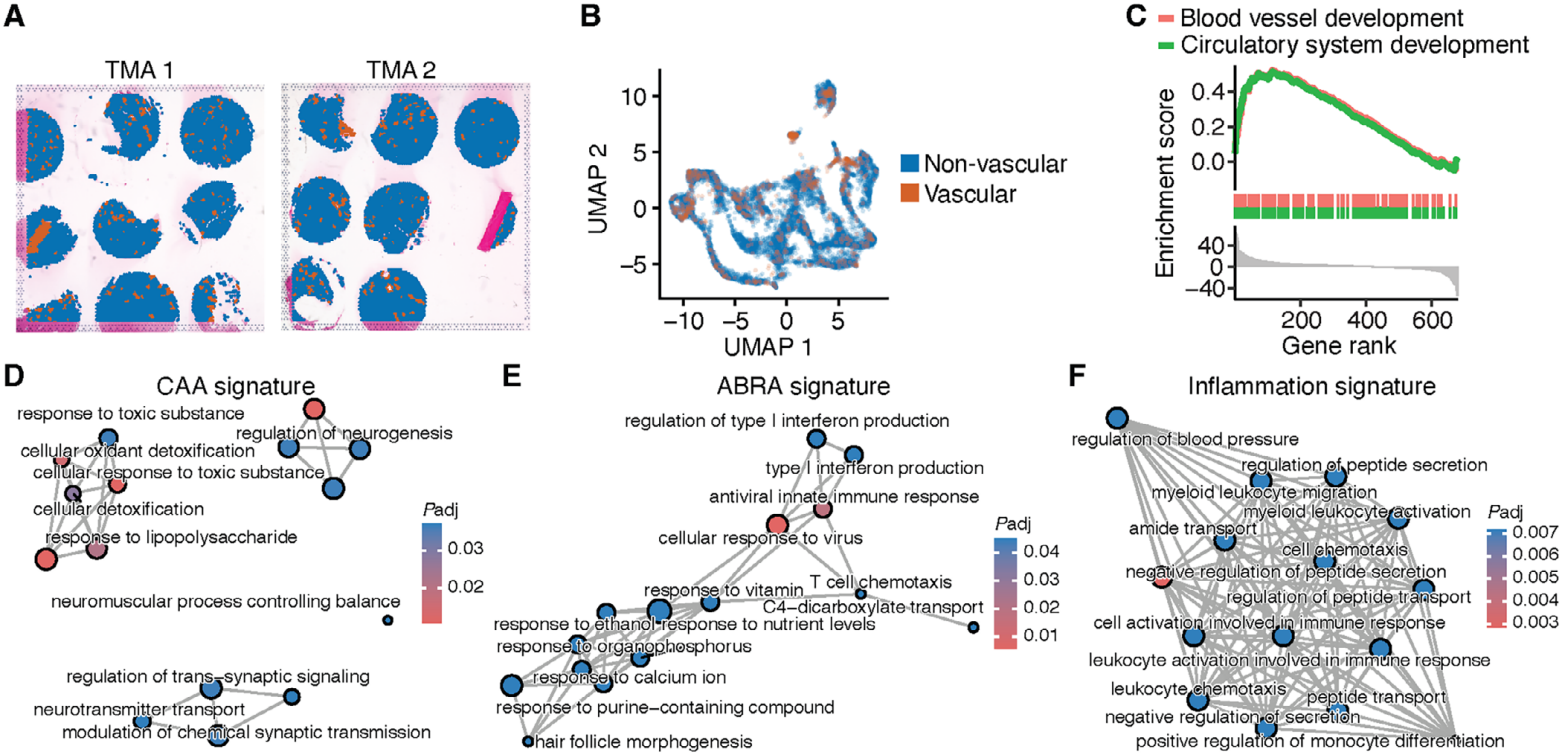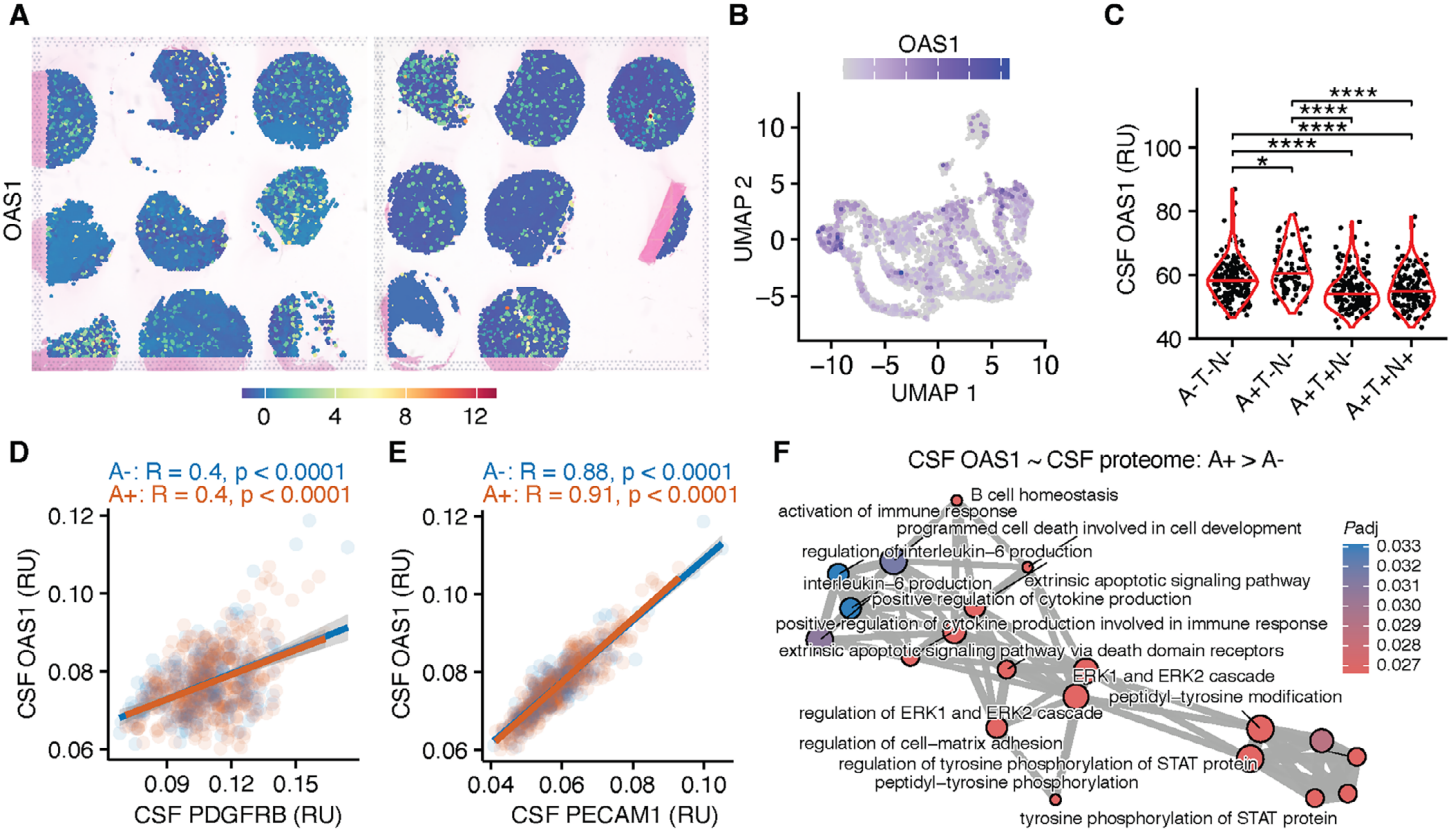# Spatial transcriptomics of cerebral amyloid angiopathy and amyloid‐β related angiitis

**DOI:** 10.1002/alz70855_104930

**Published:** 2025-12-24

**Authors:** Marcel Seungsu Woo, Alexandros Hadjilaou, Lukas Raich, Matthias Dottermusch, Björn Rissiek, Manuel A. Friese, Serge Gauthier, Pedro Rosa‐Neto, Tim Magnus, Markus Glatzel

**Affiliations:** ^1^ University Medical Center Hamburg‐Eppendorf, Hamburg, Germany; ^2^ University Medical Center Hamburg‐Eppendorf, Hamburg, Hamburg, Germany; ^3^ University Medical Center Hamburg‐Eppendorf, Department of Psychiatry, Hamburg, Hamburg, Germany; ^4^ University Medical Center Hamburg‐Eppendorf, Institute of Neuropathology, Hamburg, Hamburg, Germany; ^5^ University Medical Center Hamburg‐Eppendorf, Department of Neurology, Hamburg, Hamburg, Germany; ^6^ University Medical Center Hamburg‐Eppendorf, Institute of Neuroimmunology and Multiple Sclerosis, Hamburg, Hamburg, Germany; ^7^ Translational Neuroimaging Laboratory, The McGill University Research Centre for Studies in Aging, Montréal, QC, Canada; ^8^ Department of Neurology and Neurosurgery, and Department of Psychiatry, McGill Centre for Studies in Aging, McGill University, Montreal, QC, Canada; ^9^ McGill University, Montreal, QC, Canada; ^10^ Department of Neurology and Neurosurgery, McGill University, Montréal, QC, Canada; ^11^ Montreal Neurological Institute, Montreal, QC, Canada

## Abstract

**Background:**

The removal of amyloid‐β (Aβ) by antibodies has revolutionized the treatment landscape of Alzheimer's disease (AD). Vascular Aβ has been implicated in the emergence of amyloid‐related imaging abnormalities (ARIA). A better understanding of the effect of Aβ on blood vessels is required to find new biomarkers and treatments for ARIA.

**Method:**

We generated tissue microarrays of brain biopsies of 4 controls, 3 primary CNS vasculitis (PCNVS), 4 cerebral amyloid angiopathy (CAA), and 5 amyloid‐β‐related angiitis (ABRA) patients and performed histopathology and Visium 10x spatial RNA‐sequencing. We performed clustering, compared the spots that contained blood vessels between the 4 conditions and used gene ontology analyses to define disease‐specific signatures. Last, we tested whether the inflammatory blood vessel signatures could be identified in CSF SOMAscan proteomics of 173 A‐T‐N‐, 82 A+T‐N‐, 164 A+T+N‐, and 144 A+T+N+ individuals of the AD neuroimaging initiative (ADNI). Correlation analyses in A+ and A‐ individuals of the inflammatory signature with more than 7000 proteins were performed.

**Results:**

Tissue microarrays allowed a scalable approach for spatial transcriptomics and histology. CAA and ABRA patients showed a strong vascular Aβ accumulation but only ABRA patients had additional T cell infiltrates and stronger blood brain barrier leakage. Spatial transcriptomics allowed the deconvolution of spots that contained different parenchymal cell types like neurons, astrocytes or endothelial cells. Comparing the blood vessels between the conditions revealed a CAA‐specific signature that was characterized by cellular detoxification. PCNVS and ABRA showed an overlapping inflammatory signature, however, the blood vessels from ABRA patients showed a type I interferon response that was absent in CAA and PCNVS. Using OAS1 as a biomarker for the type I interferon response, we identified strong correlations between OAS1 and endothelial cell biomarkers in the CSF of ADNI participants. In contrast, OAS1 was stronger associated with the activation of the interferon response and cell death pathways in A+ than in A‐ participants.

**Conclusion:**

We identified Aβ‐specific inflammatory blood vessel signatures in brain biopsies and the CSF of an AD continuum cohort. This study will help to prioritize biomarkers and treatments for Aβ‐induced vascular pathologies and potentially ARIA.